# 511. Novel Dressing with Improved Application/Removal Demonstrates Comparable MRSA Control to Antimicrobial Foam in Rat Burns

**DOI:** 10.1093/jbcr/irag033.170

**Published:** 2026-04-06

**Authors:** Lian Daniel N Manalo, Abigail Bruhm, Kai V Myree Lindsey, Eriks Ziedins, Casey Meretta, Lauren T Moffatt, Jeffrey W Shupp, Bonnie C Carney

**Affiliations:** The Burn Center at MedStar Washington Hospital Center, Washington, DC; The Burn Center at MedStar Washington Hospital Center, Washington, DC; The Burn Center at MedStar Washington Hospital Center, Washington, DC; Medstar Washington Hospital Center, Washington , DC; The Burn Center at MedStar Washington Hospital Center, Potomac, MD; The Burn Center at MedStar Washington Hospital Center, Washington, DC; MedStar Washington Hospital Center, Washington, DC; The Burn Center at MedStar Washington Hospital Center, Washington, DC

## Abstract

**Introduction:**

Burn injuries compromise the skin barrier, increasing infection risk which delays healing and can be life threatening. Dressings help prevent infection by protecting the wound from environmental bacteria. This study tested a novel antimicrobial spray-on dressing, composed of hydrocolloids, designed to conform to irregular skin surfaces such as hands or joints and for its ability to alleviate burn wound infection.

**Methods:**

Nineteen male Sprague Dawley rats were assigned to four groups: negative control, the novel antimicrobial spray-on dressing, silver impregnated foam dressing, and positive control. Full-thickness scald burns were created by exposing skin to 100°C water for 10 seconds. Three groups were inoculated with methicillin-resistant *Staphylococcus aureus* (MRSA) and treated with transparent film (positive control), silver impregnated foam, or novel spray-on dressings; negative controls received no bacteria or dressing. Dressings were changed on days 2 and 5. Wound biopsies, images, and bacterial cultures were collected on days 0, 2, 5, and 8. On day 8, animals were euthanized, and blood and spleen were collected for systemic cultures. Cultures were quantified by plating homogenized biopsies on mannitol salt agar and counting colony forming units (CFUs) with ImageJ. Statistical analysis was done through a 2-way ANOVA with multiple comparisons between each group at all timepoints.

**Results:**

On day 5, bacterial burden (CFU/g) was significantly higher in the positive control group (7.02 * 10^12 ± 9.65 * 10^10) compared to the foam dressing group (1.51 * 10^8 ± 2.19 * 10^8, *p*<.01) and negative control (5.26 * 10^8 ± 8.58 * 10^8, *p*<.001). The foam dressing group was comparable to the negative control, while the spray-on dressing was comparable to the positive control group, but there were no significant differences between the novel spray-on and silver impregnated foam dressings. On day 8, there was no evidence of bacteremia (all blood cultures were negative). Finally, there were no significant differences in bacterial counts in the spleen between groups.

**Conclusions:**

The spray-on dressing did not significantly differ from the antimicrobial foam dressing, indicating comparable efficacy in controlling bacterial loads in MRSA-infected burn wounds.

**Applicability of Research to Practice:**

Spray-on dressings offer a promising option for burn wound infection management in anatomically complex areas where traditional dressings are challenging. Their conformability may improve clinical outcomes and patient comfort compared to existing dressings.

**Funding for the study:**

This work was funded in part by Ionic Pharmaceuticals and the SBIR program (R44GM125412). This work was funded in part by award number KL2TR001432 from the National Center for Advancing Translational Science (NCATS/NIH).

